# Prediction using T2‐weighted magnetic resonance imaging‐based radiomics of residual uterine myoma regrowth after high‐intensity focused ultrasound ablation

**DOI:** 10.1002/uog.26053

**Published:** 2022-11-01

**Authors:** Y. Zhou, J. Zhang, J. Chen, C. Yang, C. Gong, C. Li, F. Li

**Affiliations:** ^1^ State Key Laboratory of Ultrasound in Medicine and Engineering, College of Biomedical Engineering Chongqing Medical University Chongqing China; ^2^ Chongqing Key Laboratory of Biomedical Engineering Chongqing Medical University Chongqing China; ^3^ Key Laboratory of Biorheological Science and Technology of the Ministry of Education Chongqing University Chongqing China

**Keywords:** high‐intensity focused ultrasound, magnetic resonance imaging, prediction model, radiomics, uterine myoma

## Abstract

**Objectives:**

To develop and evaluate magnetic resonance imaging (MRI)‐based radiomics models for predicting residual myoma regrowth within 1 year after high‐intensity focused ultrasound (HIFU) ablation of uterine myomas.

**Methods:**

A retrospective analysis of residual myoma regrowth within 1 year was performed on 428 myomas in 339 patients who were diagnosed with uterine myoma and treated with HIFU ablation in two hospital centers. In total, 851 radiomics features were extracted from T2‐weighted images (T2WI) obtained 1 day after HIFU ablation, and the least absolute shrinkage and selection operator in the training cohort (*n* = 243) was employed to select radiomics features. Support vector machines were adopted to develop radiomics, clinicoradiological and combined radiomics–clinical models to predict residual myoma regrowth, defined as an increase in residual myoma volume of > 10% between that at day 1 post HIFU and that at follow‐up MRI within 1 year. These models were validated in both internal (*n* = 81) and external (*n* = 104) test cohorts. The predictive performance and clinical application of these models were assessed using receiver‐operating‐characteristics‐curve analysis, the area under the curve (AUC) and decision‐curve analysis.

**Results:**

The AUCs of the T2WI‐based radiomics prediction model in the internal and external test cohorts were 0.834 (95% CI, 0.747–0.920) and 0.801 (95% CI, 0.712–0.889), respectively, and those of the clinicoradiological model were 0.888 (95% CI, 0.816–0.960) and 0.912 (95% CI, 0.851–0.973), respectively. The combined model had better predictive performance than either the radiomics or the clinicoradiological model, with AUC values of 0.922 (95% CI, 0.857–0.987) and 0.930 (95% CI, 0.880–0.980) in the internal and external test cohorts, respectively. Decision‐curve analysis also indicated that application of the combined model has clinical value, this model achieving more net benefits than the other two models.

**Conclusion:**

T2WI‐based radiomics features can predict effectively the occurrence of residual myoma regrowth within 1 year after HIFU ablation of uterine myomas, which serves as an accurate and convenient reference for clinical decision‐making. © 2022 The Authors. Ultrasound in Obstetrics & Gynecology published by John Wiley & Sons Ltd on behalf of International Society of Ultrasound in Obstetrics and Gynecology.

**CONTRIBUTION**

**What are the novel findings of this work?**

T2‐weighted magnetic resonance imaging (T2WI)‐based radiomics parameters can predict regrowth of residual uterine myomas within 1 year after high‐intensity focused ultrasound (HIFU) treatment.

**What are the clinical implications of this work?**

Accurate prediction of residual myoma regrowth is critical for the early customization of chronic disease management plans after HIFU ablation in patients with uterine myomas. T2WI‐based radiomics may have a role in this regard.

## INTRODUCTION

High‐intensity focused ultrasound (HIFU) is well established and widely used in the treatment of uterine myomas. By analyzing patients' magnetic resonance imaging (MRI) data, Liu *et al*.^1^ reported that future reintervention was related closely to regrowth of residual myomas. In the postoperative follow‐up period, residual myomas are associated with lower mortality compared with residual malignant tumors, such as uterine sarcomas^2^. However, if the residual myoma tissue has a rich blood supply, regrowth may occur, which is the main reason for increase in tumor volume and recurrence of clinical symptoms after HIFU ablation of uterine myoma^1^, ^3^.

MRI provides high soft‐tissue resolution without exposure to radiation, making it an ideal tool for monitoring uterine myomas after HIFU ablation. However, the high cost, long scanning time and potential side effects of gadolinium‐based contrast agents used in MRI examination limits its use in follow‐up^4^. Reliable methods for prediction of regrowth of residual myomas would facilitate targeted disease management, potentially extending the duration of uterine myoma volume reduction, reducing clinical symptoms postoperatively and improving the patient's quality of life after surgery. Currently, there is no method to predict regrowth of residual myomas.

Radiomics is an emerging field that extracts from medical images a large number of quantitative features using data characterization algorithms and mathematical tools^5^, ^6^. On T2‐weighted imaging (T2WI), there is good contrast resolution between uterine myomas and the surrounding tissue. T2WI‐based radiomics studies of uterine lesions have been able to differentiate between benign and malignant uterine tumors^7^ and to predict effectively the non‐perfused volume ratio (NPVR) of myomas after HIFU^8^. Whether T2WI‐based radiomics analysis can help to provide a prognosis for uterine myomas has not been reported. In this study, we aimed to analyze the heterogeneity of lesions after ablation and the association with regrowth of residual myomas by extracting T2WI radiomics features 1 day after HIFU ablation and developing a machine‐learning model to predict prognosis.

## methods

This retrospective study was conducted with the approval of the Ethics Committees of Chongqing Haifu Hospital (IRB‐2022002) and the First Affiliated Hospital of Chongqing Medical University (IRB‐2006016). The requirement for informed consent was waived.

### Patients

**Figure 1:**
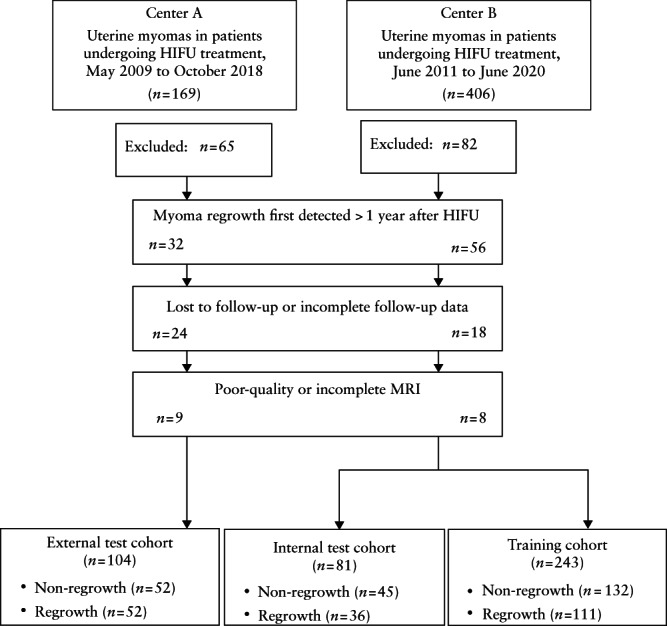
Flowchart summarizing patient enrolment process and study cohorts. HIFU, high‐intensity focused ultrasound; MRI, magnetic resonance imaging.

We obtained data retrospectively from the hospital databases for patients diagnosed with one or more uterine myomas based on imaging findings combined with clinical symptoms who were treated with HIFU ablation at the First Hospital of Chongqing Medical University between May 2009 and October 2018 (*n* = 104) and Chongqing Haifu Hospital between June 2011 and June 2020 (*n* = 324). Inclusion criteria were: (1) premenopausal and aged ≥ 18 years; (2) symptoms related to uterine myomas; (3) safe acoustic channel (i.e. without intestinal tissue), with clear visibility of myomas under ultrasound guidance; (4) remained conscious during surgery; (5) MRI performed before and after surgery; and (6) myoma maximum diameter ≥ 3 cm on pre‐HIFU MRI. Exclusion criteria were: (1) myoma regrowth detected after more than 1 year; (2) poor image quality or incomplete image data; (3) contraindication to MRI examination; (4) acute pelvic inflammation; and (5) acoustic channel scarring in the lower abdomen, resulting in significant acoustic attenuation. We also carried out a prospective telephone follow‐up survey of all included cases, to ask whether reintervention had been undertaken. Figure 1 summarizes the study cohorts and Figure S1 illustrates the study workflow.

### HIFU ablation

A JC‐type focused ultrasound tumor treatment system (Chongqing Haifu Medical Technology Co., Chongqing, China), which was monitored in real‐time during surgery using a color Doppler ultrasound scanner (Mylab 70, Esaote, Genoa, Italy), was employed to treat all single and multiple uterine myomas. The therapeutic ultrasound transducer used for this procedure had a working frequency of 0.8 MHz, diameter of 20 cm, power of up to 400 W, physical focal field of 1.5 × 1.5 × 8.0 mm, focal length of 18 cm and onboard imaging ultrasound frequency of 3.5 MHz. Before surgery, preoperative preparations including lower abdominal skin degassing and cleansing enema were completed, and a catheter was inserted in order to instil saline to control the volume of the bladder. During the operation, the patient was in a prone position on the treatment table, enabling the lower abdominal skin to be in full contact with the degassing water. The direction and angle of the ultrasound probe were adjusted by the operator to guide treatment of the target myoma. The acoustic wave emission power and dose delivery were adjusted according to both the patient's tolerance level and the change in appearance of the myoma on grayscale imaging. Fentanyl citrate (0.8–1.0 µg/kg) and midazolam maleate (0.02–0.03 mg/kg) were injected intravenously every 30–40 min to maintain conscious sedation during surgery, with blood pressure, heart rate, oxygen saturation and respiratory rate being monitored throughout. The extent of ablation was assessed intraoperatively and immediately after surgery using an ultrasound contrast agent (SonoVue; Bracco, Milan, Italy).

### MRI

To evaluate the feasibility beforehand and the effectiveness after HIFU for uterine myoma ablation, each patient underwent pelvic contrast‐enhanced (CE) MRI prior to treatment, 1 day post‐HIFU and in a follow‐up examination, using a 3‐Tesla (T) MRI scanner (GE Healthcare, Milwaukee, WI, USA) or a Magnetom 1.5‐T MRI system (Umr570, United Imaging Company, Shanghai, China). T1‐weighted imaging, T2WI and CE MRI sequences were used to acquire sagittal, cross‐sectional and coronal images, respectively. Acquisition parameters are specified in Table 1.

**Table 1:** Magnetic resonance imaging (MRI) scan parameters

MRI type	Repetition time (ms)	Echo time (ms)	Number of excitations	Field of view (cm × cm)	Matrix size (mm × mm)	Slice thickness (mm)	Slice gap (mm)	Imaging planes
T1‐weighted	175/214	1.8/10	1/1.3	40 × 28/25.2 × 36	320 × 224/320 × 70	5/5	1.5/1.0	T
T2‐weighted	4060/5300	100/88	3/2	22.4 × 28/24 × 24	288 × 224/320 × 75	5/5	1.5/1.0	T, S
Contrast‐enhanced MRI	4.2/3.94	1.9/1.84	0.72/1	38 × 30.4/35 × 28	320 × 224/288 × 75	4/2.5	0/0.5	T, S, C

Parameters are presented as those used by the First Affiliated Hospital of Chongqing Medical University/those used by Chongqing Haifu Hospital. C, coronal; S, sagittal; T, transverse.

### Clinicoradiological features, residual myoma regrowth criteria and image segmentation

All MRI information for this analysis was retrieved retrospectively from the Picture Archiving and Communication System (PACS) in Digital Imaging and Communications in Medicine (DICOM) format. Clinicoradiological features, obtained from MRI examinations before and 1 day after HIFU, included: patient age, duration of follow‐up, International Federation of Gynaecology and Obstetrics stage^9^, T2WI type, degree of blood supply, number of myomas detected and treated per patient, maximum thickness and basal extent of residual myomas, NPVR, postoperative NPV, postoperative residual myoma volume, thickness of the abdominal wall fat layer and distance from the center of the myoma to the body surface.

**Figure 2:**
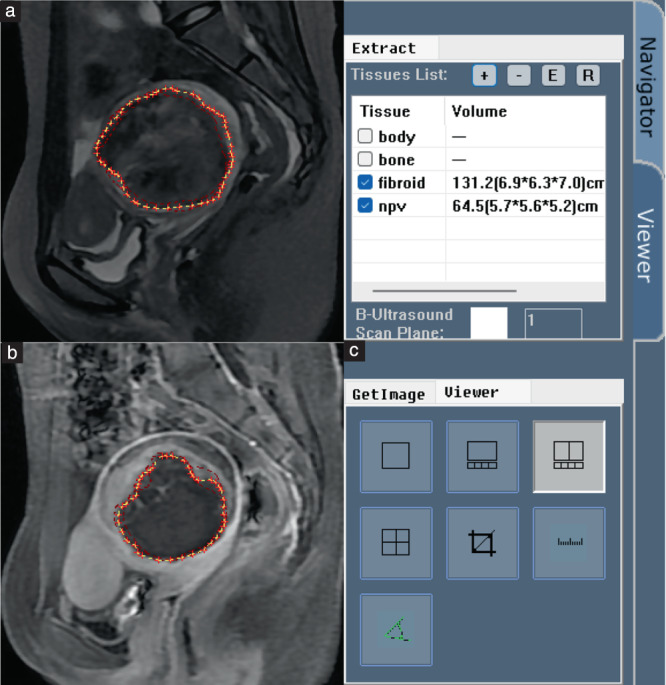
MicroSea 3D Image Processing Software was used to determine myoma volume and volume of non‐perfused areas using a layer‐by‐layer process. (a) Myomas were traced manually on T2‐weighted magnetic resonance (MR) images. (b) Non‐perfused areas were traced on contrast‐enhanced MR images. (c) Software calculated the three‐dimensional volume.

Using MicroSea 3D Image Processing Software (Chongqing MicroSea Software Development Co., Ltd., Chongqing, China), an abdominal radiologist with 5 years' experience in pelvic radiography (Reader 1) evaluated the clinical and radiological features and traced the outlines layer‐by‐layer on MR images, to measure the three‐dimensional (3D) uterine myoma volume and non‐perfused volume (NPV) (Figure 2). The NPVR (%) was defined as NPV as a percentage of myoma volume, and the residual myoma volume (RMV) was calculated by subtracting NPV from myoma volume. This strategy was also applied to volume measurements during follow‐up. An increase in RMV at the follow‐up MRI exam of greater than 10% from that in the immediate postoperative period (1 day post HIFU) was set as the criterion for identifying residual myoma regrowth^1^, and open‐source software (ITK‐SNAP v3.8.0, www.itksnap.org) was used to segment regions of interest (ROIs) on T2WI obtained within 1 day post HIFU, layer‐by‐layer along the edges of the myoma. The feature identification and image segmentation outlined above were validated by another radiologist (Reader 2) with 16 years' experience in pelvic radiography; in the case of disagreement, Reader 2 retraced the ROI and this was used as the final decision.

### Image preprocessing and radiomics feature extraction

Before radiomics feature extraction, T2WI underwent three preprocessing steps to minimize the variance introduced by different scanners, scanning schemes and acquisition artifacts. First, the ‘N4ITK’ bias field correction algorithm was applied to all images to reduce image artifacts and improve the grayscale distribution^9^. Next, all images were normalized by calculating *Z*‐scores to obtain a standard normal distribution of the image intensity. Finally, resampling was achieved by setting the voxel size to 1 × 1 × 1 mm^3^ with B‐spline interpolation. For more details on image processing, see Table S1.

Radiomics features for each ROI were extracted from T2WI images obtained 1 day after HIFU using Pyradiomics v.3.0 (https://pypi.org/project/pyradiomics/), following the Image Biomarker Standardisation Initiative (IBSI) guidelines^10^. Extracted features included 851 features of shape (3D), first‐order statistics, gray‐level co‐occurrence matrix (GLCM), gray‐level size zone matrix (GLSZM), gray‐level run‐length matrix (GLRLM), neighboring gray tone difference matrix (NGTDM) and gray‐level dependence matrix (GLDM) and wavelets. Details are presented in Table S2.

### Reproducibility analysis of radiomics features and feature selection

To assess the reproducibility of the radiomics features extracted from the ROI, Reader 2 selected 30 images arbitrarily, on which both Readers 1 and 2 performed ROI segmentation prior to the radiomics feature extraction process. Intraclass correlation coefficients (ICCs) were used to assess the reproducibility of the radiomics features extracted from all ROIs that were traced by the two radiologists, and any feature with ICC ≥ 0.8 was considered reliable.

All radiomics features with ICC ≥ 0.8 were standardized by *Z*‐score. The best radiomics features in the training cohort were identified and selected using the least absolute shrinkage and selection operator (LASSO) method, and the optimal value of the parameter λ was determined based on a 10‐fold cross‐validation. Individual radiomics scores (Rad‐scores) were calculated from a linear combination of each feature along with its coefficient weighting according to the non‐zero coefficient features selected by LASSO.

### Model construction and evaluation

The clinicoradiological model was constructed by combining clinical and radiological features that showed statistical differences in the training cohort: patient age, T2WI signal intensity of myomas (T2WI type), degree of myoma blood supply, NPVR, RMV, maximum distance from surface to center of myomas, FIGO stage, maximum thickness of residual myomas and their basal distribution range. Additionally, follow‐up time is an important feature in predicting myoma regrowth and was included for construction of clinicoradiological and combination models. A radiomics model was constructed using T2WI radiomics features. A radiomics–clinical model was constructed by combining radiomics features with clinicoradiological features.

We employed support vector machines (SVM) to build the model (Python scikit‐learn environment, version 0.21.3; https://scikit‐learn.org/stable/index.html). In the training cohort, 10‐fold cross‐validation and grid search were applied to select optimal model parameters and kernel functions. The receiver‐operating‐characteristics (ROC) curve was generated to evaluate whether the model had the ability to predict the regrowth of residual myomas, with validation carried out on the model in both internal and external test cohorts. In addition, the predictive performance of the model in different cohorts was evaluated using the area under the ROC curve (AUC). To obtain the AUC 95% CI, we applied non‐parametric bootstrap sampling to perform the calculation 2000 times.

### Evaluation of clinical application

Decision‐curve analysis was performed to assess whether the prediction model would help in the development of clinical treatment strategies by calculating the net benefit of the model at different threshold probabilities in the three cohorts. The best prediction model was determined by the AUC value and used to predict residual myoma regrowth in patients on days 30, 90, 180, 270 and 360.

### Statistical analysis

Continuous data are presented as the mean ± SD or median and interquartile range (IQR), depending on whether the data were distributed normally. We applied the χ^2^ test or Fisher's exact test to compare differences in categorical variables and the Mann–Whitney *U*‐test to compare differences in continuous variables. Categorical variables were described by frequency and rate, and a two‐sided test with *P* < 0.05 indicated statistical significance.

## Results

There was residual myoma regrowth after HIFU ablation in 199/428 (46.5%) of myomas: 111/243 (45.7%) in the training cohort, 36/81 (44.4%) in the internal test cohort and 52/104 (50.0%) in the external test cohort. Baseline clinical and radiological features are presented in Table 2. There were significant differences in patient age, T2WI type, blood supply, maximum thickness and basal extent of residual myomas, NPVR, and postoperative RMV in all three datasets (training, internal test and external test sets). Patients were younger in the regrowth group compared with in the non‐regrowth group (*P* < 0.05). The proportions of T2WI Types III and IV were significantly higher in the regrowth group than in the non‐regrowth group, whereas Type I was predominant in the non‐regrowth group (*P* < 0.05). The proportion of the regrowth group with abundant blood supply was significantly higher than that in the non‐regrowth group (*P* < 0.001). The regrowth group had greater maximum thickness, wider basal distribution range and larger volume of residual myomas compared with the non‐regrowth group (*P* < 0.001). The NPVR was significantly lower in the regrowth group than in the non‐regrowth group (*P* < 0.001).

**Table 2:** Baseline characteristics, including clinical and radiological features, in study population of patients with uterine myomas treated with high‐intensity focused ultrasound (HIFU) ablation

Characteristic	Regrowth	Non‐regrowth	Z‐statistic	*P* *
*n* (training/internal validation/external validation cohorts)	111/36/52	132/45/52		
Patient's age (years)				
Training	39 (33.5–43)	41 (35–45)	–2.408	0.016
Internal	34 (30.5–40)	42 (36–45)	–3.257	0.001
External	34 (29–39)	37.5 (33–42)	–2.138	0.032
Follow‐up time (days)†				
Training	200.0 (149.0–277.5)	201.5 (138.5–272.0)	–0.420	0.674
Internal	194.0 (137.0–227.0)	249.0 (192.0–377.0)	–3.241	0.001
External	178.0 (105.0–32.0)	119.5 (92.3–256.8)	–1.726	0.084
*Before HIFU*				
FIGO stage				
Training	4.0 (4.0–6.0)	4.0 (4.0–5.0)	–2.410	0.016
Internal	5.5 (4.0–6.0)	4.0 (3.0–4.0)	–3.099	0.002
External	4.5 (4.0–6.0)	4.0 (4.0–6.0)	–1.508	0.132
T2WI type (I/II/III/IV) (*n*)‡				
Training	46/7/37/21	84/12/25/11	–3.841	< 0.001
Internal	13/4/11/8	26/8/10/1	–2.696	0.004
External	16/13/16/7	33/10/8/1	–3.614	< 0.001
Degree of blood supply (low or absent/moderate/rich) (*n*)				
Training	8/47/56	43/67/22	–6.371	< 0.001
Internal	1/19/16	19/18/8	–3.961	< 0.001
External	0/39/13	15/33/4	–4.210	< 0.001
Number of myomas (per patient)				
Training	2.0 (1.0–≥ 5.0)	2.0 (1.0–≥ 5.0)	–1.120	0.236
Internal	1.0 (1.0–2.5)	2.0 (1.0–5.0)	–0.929	0.259
External	1.0 (1.0–5.0)	2.0 (1.0–5.0)	–1.282	0.200
Abdominal fat thickness (cm)				
Training	1.29 (0.94–1.85)	1.37 (0.96–1.77)	–0.504	0.614
Internal	1.14 (0.68–1.73)	1.37 (1.02–1.85)	–1.402	0.161
External	1.27 (1.07–1.66)	1.52 (1.02–1.93)	–1.291	0.197
Maximum AP distance from surface to center of myomas (cm)				
Training	7.95 (6.27–10.25)	7.03 (5.74–8.67)	–2.614	0.009
Internal	6.74 (5.75–8.64)	6.93 (6.43–7.99)	–0.699	0.485
External	6.90 (5.71–9.34)	7.29 (6.19–9.11)	–0.731	0.465
*1 day post HIFU*				
Number of treated myomas (per patient)				
Training	2 (1 to ≥ 5)¶	2 (1 to ≥ 5)¶	–0.618	0.525
Internal	1 (1–2)	1(1–5)	–1.483	0.094
External	1 (1–3)	1 (1–4)	–0.755	0.450
Maximum thickness of residual myomas (cm)				
Training	1.14 (0.81–1.51)	0.50 (0.28–0.66)	–10.532	< 0.001
Internal	1.05 (0.84–1.70)	0.42 (0.00–0.65)	–6.442	< 0.001
External	1.21 (0.82–1.60)	0.43 (0.27–0.65)	–6.706	< 0.001
Basal distribution of residual myomas§				
Training	3 (2–4)	1 (1–2)	–9.394	< 0.001
Internal	3 (2–3.5)	1 (0–2)	–5.199	< 0.001
External	3 (2–3)	1 (1–2)	–6.081	< 0.001
NPVR				
Training	57.14 (40.52–69.83)	81.26 (69.24–89.80)	–9.344	< 0.001
Internal	64.20 (47.69–69.82)	82.93 (72.14–90.33)	–4.892	< 0.001
External	59.04 (48.63–72.53)	85.29 (69.56–95.02)	–6.095	< 0.001
Postoperative NPV (cm^3^)				
Training	40.00 (16.60–87.15)	48.00 (22.85–87.65)	–1.386	0.166
Internal	61.60 (33.25–120.00)	48.30 (20.53–103.08)	–1.511	0.131
External	36.60 (21.03–73.25)	41.55 (20.85–65.25)	–0.189	0.850
Postoperative RMV (cm^3^)				
Training	30.00 (15.58–60.45)	10.70 (4.50–22.31)	–7.458	< 0.001
Internal	41.15 (20.60–67.75)	11.20 (3.70–20.50)	–4.862	< 0.001
External	27.00 (12.55–44.58)	6.20 (2.98–14.05)	–6.225	< 0.001

Data are presented as median (interquartile range), unless stated otherwise.

* Significance indicated by *P* < 0.05 (Mann–Whitney *U*‐test).

† From 1 day post HIFU to follow‐up magnetic resonance imaging within 1 year.

‡ Myoma type according to signal intensity on pretreatment T2‐weighted imaging (T2WI): Type I: hypointense, signal intensity equal to that of skeletal muscle; Type II: isointense, signal intensity lower than that of uterine myometrium but higher than that of skeletal muscle; Type III: heterogeneous hyperintense; Type IV, homogeneous hyperintense.

§ Basal distribution of residual myomas quantified according to Zhang *et al*.^27^, based on the distribution across four equal quadrants of the original myoma distribution: 0, no residual myoma; 1, limited distribution; 2, small distribution; 3, obvious distribution; 4, extensive distribution.

¶ It was not always possible to count multiple myomas, so these were expressed as ≥ 5. AP, anteroposterior; FIGO, International Federation of Gynaecology and Obstetrics^28^; NPV, non‐perfused volume; NPVR, non‐perfused volume ratio; RMV, residual myoma volume.

### Radiomics feature selection and radiomics score (Rad‐score) calculation

Among the 851 radiomics features extracted, the interobserver consistency for their extraction from ROIs was good, with ICC ≥ 0.8 for 665 (78.1%) features. From these, 17 non‐zero coefficient features were selected by LASSO, including one GLDM feature, one GLRLM feature and 15 wavelet features.

**Figure 3:**
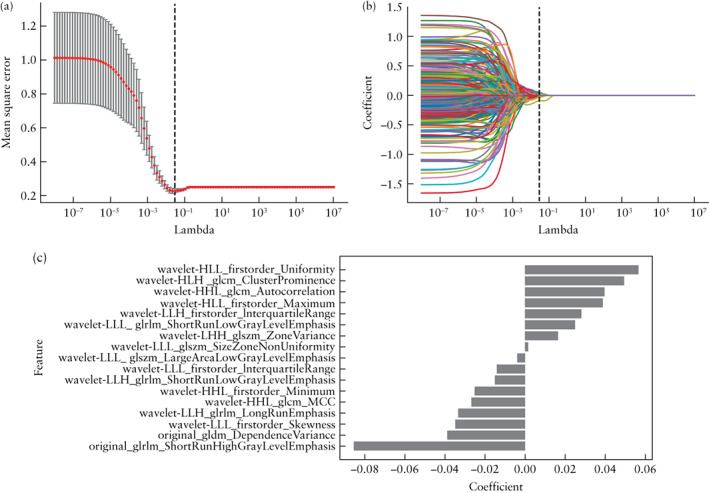
Radiomics feature selection. (a) Selection, via mean square error, of tuning parameter λ in least absolute shrinkage and selection operator (LASSO) model, on each fold of the 10‐fold cross‐test method; gray vertical lines show optimal λ values according to the corresponding minimum mean square error. (b) LASSO coefficient profiles of the 851 radiomics features, resulting in 17 non‐zero coefficient features. (c) The 17 most predictive feature subsets selected by LASSO and their correlation coefficients.

**Figure 4:**
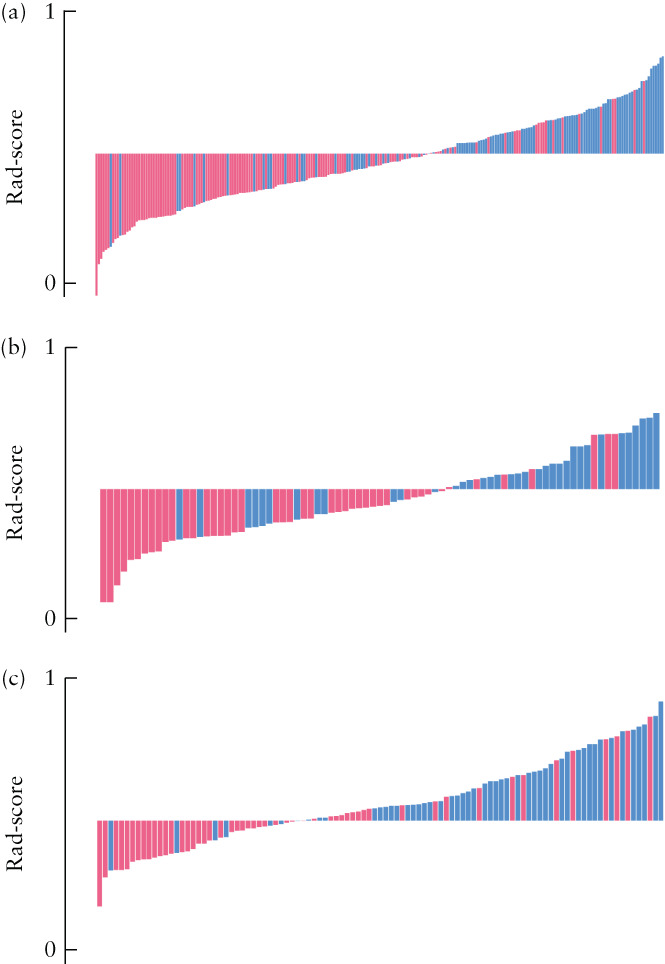
Radiomics score (Rad‐score) boxplots of the three study cohorts: (a) training cohort, (b) internal test cohort and (c) external test cohort. Red bars represent patients with residual uterine myoma non‐regrowth, and blue bars represent patients with regrowth. Rad‐score cut‐off value for prediction of myoma regrowth = 0.475.

Figure 3 illustrates the process of LASSO feature selection, as well as the selected features, with their coefficients, that were used to calculate the Rad‐score for each patient. Figure 4 shows the distribution of the Rad‐score values for each patient in the training cohort and the two test cohorts. An optimal Rad‐score cut‐off value of 0.475 was determined based on the maximum Youden index in the training‐cohort patients who had regrowing residual myomas. The significantly higher Rad‐score in patients with than in those without regrowth was confirmed in the two test cohorts, indicating that the selected radiomics features could distinguish effectively between myomas which would regrow and those which would not. The equation and statistical analyses for the Rad‐scores are presented in Appendix S1 and Table S3, respectively.

### Model evaluation

**Figure 5:**
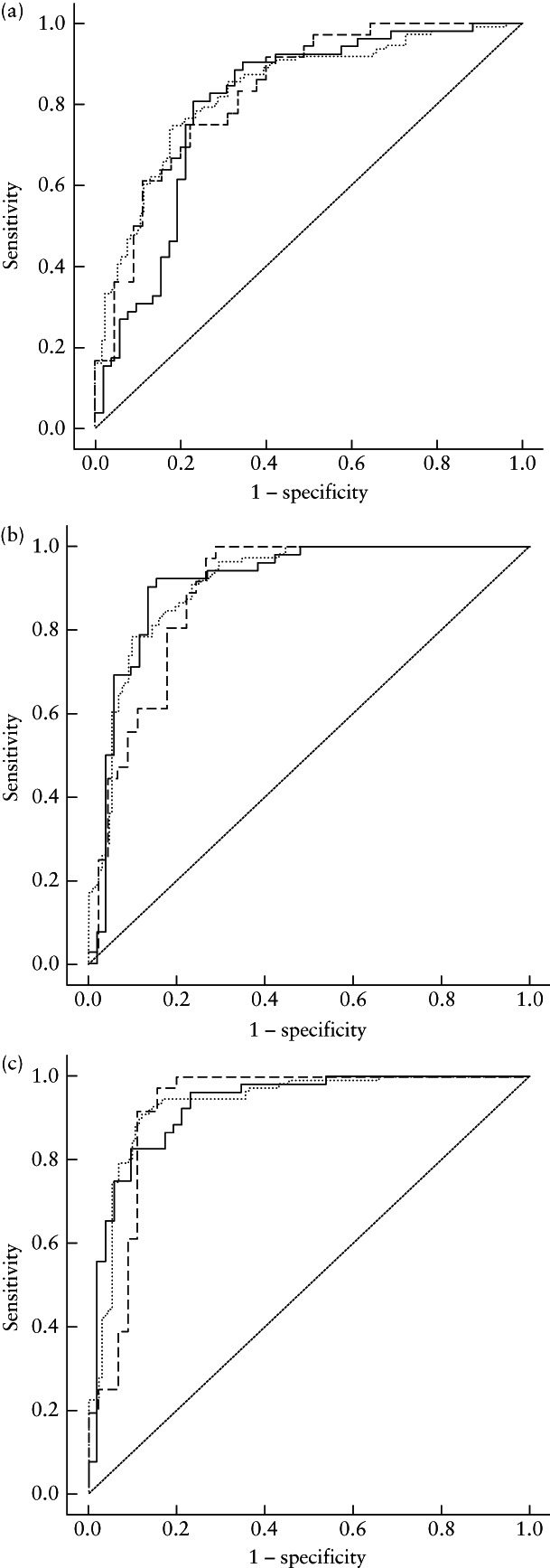
Receiver‐operating‐characteristics curves for prediction of residual uterine myoma regrowth after high‐intensity focused ultrasound ablation by: (a) radiomics model, (b) clinicoradiological model and (c) combined radiomics–clinical model. Curves are shown for training cohort (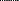), internal test cohort (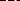) and external test cohort (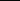).

The linear kernel was selected as the most suitable kernel function for SVM modeling. The prediction performances of the clinicoradiological model, radiomics model and combined radiomics–clinical model are presented in Table 3 and Figure 5. The radiomics model, based on the selected 17 T2WI features, showed excellent predictive performance, with AUCs of 0.834 (95% CI, 0.747–0.920) in the internal test cohort and 0.801 (95% CI, 0.712–0.889) in the external test cohort. The AUC values of the clinicoradiological model, based on patient age, T2WI signal intensity of myomas, degree of myoma blood supply, NPVR, RMV, follow‐up time, maximum distance from surface to center of myomas, maximum thickness of residual myomas and their basal distribution range, were 0.888 (95% CI, 0.816–0.960) in the internal test cohort and 0.912 (95% CI, 0.851–0.973) in the external test cohort. The model combining clinical and radiomics features had the greatest predictive validity: the AUCs of this prediction model were 0.922 (95% CI, 0.857–0.987) in the internal test cohort 0.930 (95% CI, 0.880–0.980) in the external test cohort. Individual ROC curves with 95% CIs are plotted in Figure S2.

**Table 3:** Performance of radiomics, clinicoradiological and combined radiomics–clinical models in prediction of residual uterine myoma regrowth after high‐intensity focused ultrasound ablation

Model	AUC	Sensitivity	Specificity	PPV	NPV	Accuracy
Training set (*n* = 243)						
Radiomics	0.835 (0.783–0.886)	0.788 (0.709–0.852)	0.774 (0.680–0.847)	0.818 (0.740–0.878)	0.739 (0.645–0.815)	0.782 (0.726–0.829)
Clinicoradiological	0.911 (0.874–0.947)	0.829 (0.754–0.885)	0.845 (0.756–0.906)	0.879 (0.808–0.927)	0.784 (0.694–0.854)	0.835 (0.783–0.877)
Combination	0.933 (0.901–0.965)	0.894 (0.825–0.939)	0.874 (0.794–0.927)	0.894 (0.825–0.939)	0.874 (0.794–0.927)	0.885 (0.838–0.919)
Internal test set (*n* = 81)						
Radiomics	0.834 (0.747–0.920)	0.740 (0.594–0.849)	0.742 (0.551–0.875)	0.822 (0.674–0.915)	0.639 (0.462–0.787)	0.741 (0.635–0.824)
Clinicoradiological	0.888 (0.816–0.960)	0.755 (0.608–0.862)	0.750 (0.563–0.879)	0.822 (0.674–0.915)	0.667 (0.489–0.809)	0.753 (0.649–0.835)
Combination	0.922 (0.857–0.987)	0.869 (0.731–0.946)	0.857 (0.689–0.946)	0.889 (0.752–0.958)	0.833 (0.665–0.930)	0.864 (0.771–0.924)
External test set (*n* = 104)						
Radiomics	0.801 (0.712–0.889)	0.868 (0.711–0.951)	0.712 (0.586–0.814)	0.635 (0.489–0.760)	0.904 (0.782–0.964)	0.769 (0.679–0.840)
Clinicoradiological	0.912 (0.851–0.973)	0.767 (0.637–0.862)	0.864 (0.719–0.943)	0.885 (0.759–0.952)	0.731 (0.587–0.840)	0.808 (0.721–0.873)
Combination	0.930 (0.880–0.980)	0.891 (0.756–0.959)	0.810 (0.682–0.897)	0.789 (0.649–0.885)	0.904 (0.782–0.964)	0.846 (0.764–0.904)

Values in parentheses are 95% CI. AUC, area under the receiver‐operating‐characteristics curve; NPV, negative predictive value; PPV, positive predictive value.

### Clinical application

**Figure 6:**
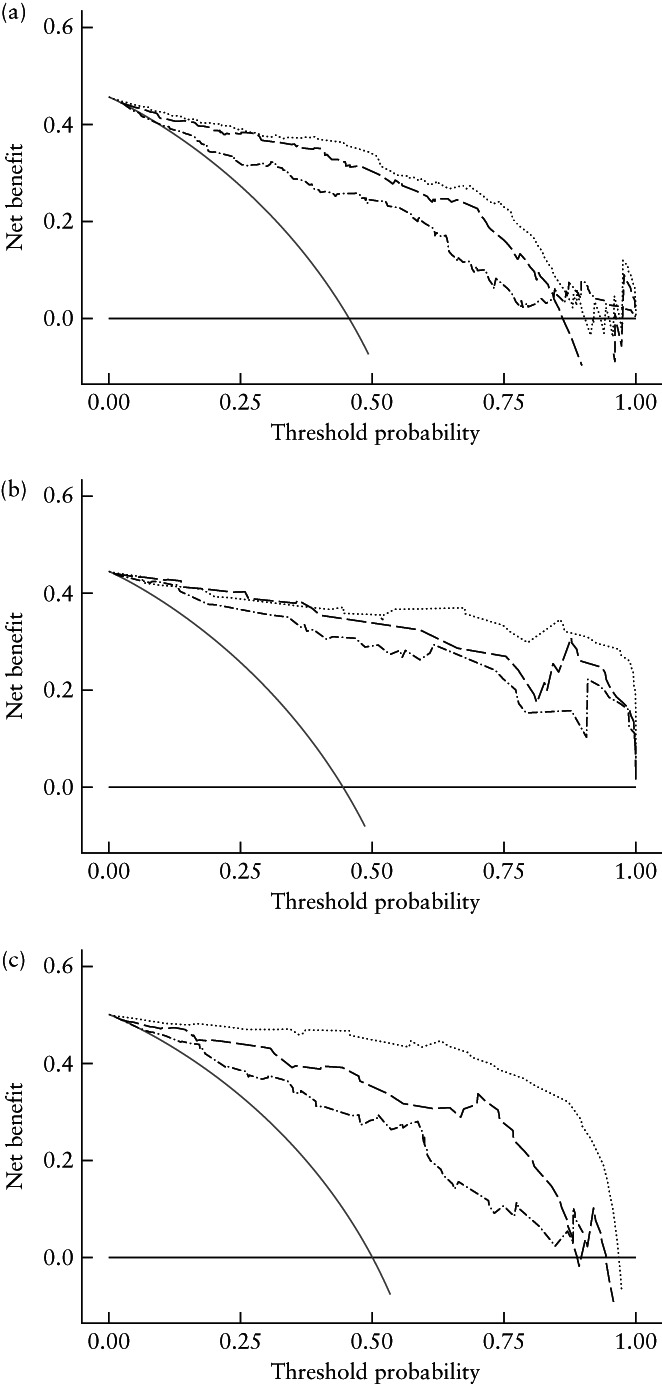
Decision‐curve analysis of radiomics, clinicoradiological and combined models for: (a) training cohort, (b) internal test cohort and (c) external test cohort. Gray and black lines represent all patients receiving the ‘all treatment’ or ‘no treatment’ strategy, respectively, and curves represent the radiomics model (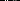), clinicoradiological model (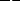) and combined radiomics–clinical model (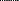).

Decision‐curve analysis can represent clinical utility based on the determined net benefits. In this study, the strategies of ‘treat all’ (reintervention therapy for all myomas, whether regrowing or not) or ‘no treatment’ (no reintervention therapy for any myoma) were interpreted as the net benefit to patients who were predicted to have regrowth or non‐regrowth of residual myomas. The results of the decision‐curve analysis indicated that the combined radiomics–clinical model achieved greater net benefit across the majority of the range of threshold probabilities than did the radiomics model, the

clinicoradiological model, the treat‐all strategy and the treat‐none strategy (Figure 6).

**Figure 7:**
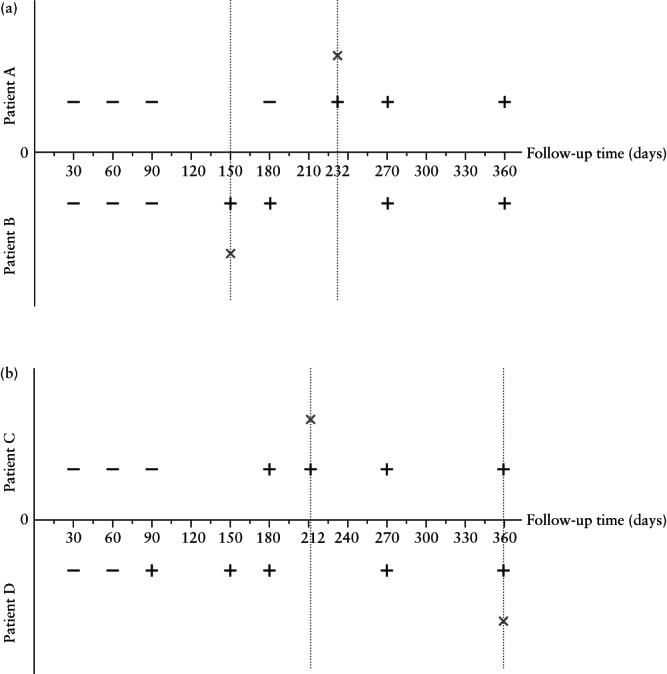
Combined radiomics–clinical model was used to predict outcome of four cases (Patients A–D, regrowth group) of residual myomas at different timepoints (30, 90, 180, 270 and 360 days post high‐intensity focused ultrasound treatment). + indicates that predicted result is regrowth, – indicates that predicted result is non‐regrowth and × indicates the point at which follow‐up magnetic resonance imaging examination was performed, in patients with myoma regrowth.

Applying the combined radiomics–clinical model to predict residual myoma regrowth on days 30, 90, 180, 270 and 360 (Figure 7), we obtained rates of correct prediction of residual myoma regrowth of 83.3% in the internal test cohort and 90.4% in the external test cohort, suggesting that the combined model is a promising tool for predicting residual myoma regrowth at different timepoints following HIFU.

### Reintervention follow‐up survey

During a median follow‐up time for the reintervention survey of 43 (IQR, 24–71) months, 323 of the 339 patients were included, while 16 patients were lost to follow‐up. The survey findings suggested that 65/323 (20.1%) patients underwent reintervention after HIFU ablation. Of these 65 cases, 21 (32.3%) underwent HIFU ablation, 36 (55.4%) myomectomy and three (4.6%) hysterectomy, three (4.6%) were treated with medication and there were two (3.1%) other cases. In addition, at the time of our follow‐up survey, nine (13.8%) patients with residual myoma regrowth were considering what type of reintervention to take. The interval between surgery and the occurrence of reintervention was 18 (IQR, 12–36) months.

Among the 65 patients who underwent reintervention, 84.6% (*n* = 55) had residual myoma regrowth. When residual myoma regrowth occurred, the subsequent reintervention rate was 40.4% (55/136), while the reintervention rate was only 5.3% (10/187) when there was no occurrence of regrowth.

## Discussion

Using our combined model to predict a patient's prognosis may help to address the problem that follow‐up after HIFU ablation generally relies on expensive MRI examinations. The overall reintervention rate in our study population was 20.1%, and 84.6% of these patients had residual myoma regrowth. Adding to these the nine patients with myoma regrowth who, at the time of follow‐up, had not yet reached a decision regarding reintervention, the percentage of residual myoma regrowth in those undergoing reintervention was as high as 86.5%. The reintervention rate in patients with myoma regrowth was 40.4%, much higher than the 5.3% reintervention rate in patients without regrowth. Thus, regrowth of residual myomas is the dominant risk factor for reintervention after HIFU ablation of myomas. Effectively predicting such factors can aid in predicting the patient's prognosis and enable initiation of appropriate clinical intervention, such as continued follow‐up, endocrine therapy, treatment for overall physical and mental wellbeing^11^, repetition of HIFU or myoma resection, in order to avoid or limit adverse effects.

We found that younger women were more likely to experience residual myoma regrowth, which may be related to the fact that uterine myomas are hormone‐dependent benign tumors. This is consistent with the findings of Gorny *et al*.^12^, who suggested that younger women are more likely to undergo reintervention after MR‐guided HIFU treatment of myomas. We found that patients with residual myoma regrowth predominantly had myomas with high signal intensity (T2WI Type III–IV), and the proportion of patients with abundant blood supply was significantly higher in those with than in those without myoma regrowth. This is probably because the areas that tend to remain non‐ablated during HIFU are usually those that are rich in blood supply^13^ or smooth‐muscle cells^14^, ^15^, and these characteristics are fundamental to residual myoma regrowth^16^. These characteristics suggest that, during HIFU ablation of uterine myomas, the more difficult it is to ablate the myoma, the more likely it is to regrow. The maximum thickness, basal distribution range and volume of residual myomas can be used to describe their morphological characteristics.

Unlike the radiomics model, which extracted MRI features automatically, in the clinicoradiological model, MRI features were selected and measured manually. However, identification of MRI features is subjective, and both feature selection and accurate measurement of features rely on the physician's clinical experience. Although the radiomics model had a weaker predictive performance than did the clinicoradiological model, it was based only on single‐sequence T2WI, without CE. Our results indicate that radiomics can identify effectively shape, texture and grayscale features of residual myomas on T2WI after HIFU ablation. Future work should assess the effect of including multiple MRI sequences in the radiomics model to further improve prediction accuracy.

In recent years, radiomics has been emerging as a tool capable of extracting ‘hidden’ data from medical images. It has been used to quantify tumor heterogeneity by analyzing the spatial arrangement of imaging voxels with variations in signal intensity, assisting in medical decision‐making^17^. Radiomics has been employed in the field of uterine myoma research, for example for the differentiation of benign and malignant tumors and the prediction of HIFU NPVR^8^, ^18^ ^,^ ^19^. However, no study has explored the application of radiomics in predicting prognosis after HIFU ablation of uterine myomas. In our study, radiomic features of different categories were incorporated into the residual myoma regrowth prediction model. The GLRLM feature, GLRLM_SRHGLE (Short Run High Gray Level Emphasis), showed the greatest significance at the time of modeling in the LASSO feature selection results. This feature represents the distribution of voxel homogeneity by measuring the distribution of short‐range run lengths for higher grayscale values; these gradually decreased as the spatial inhomogeneity of the voxel alignment increased^20^, ^21^. Wavelet features can further reflect the spatial heterogeneity of tumor tissue on multiple levels to provide more detailed information on tumor biology and supplement visual features. Higher Rad‐scores also indicated greater lesion heterogeneity and increased risk of residual myoma regrowth, whereas lower scores indicated more homogeneous lesions and a better prognosis, consistent with previous study findings on cervical cancer^22^. The feature variability detected by radiomics in patients with residual myomas may be an early indicator of continued myoma progression, enabling the identification of heterogeneity characteristics, i.e. the radiomics features that identify heterogeneous changes in myomas, at the microscopic level^23^, ^24^. Furthermore, quantitative measurements of specific tissue areas can provide additional information on subtle differences in tissue structure, including areas of necrosis, cell proliferation and neoangiogenesis^25^, ^26^, that relate to intrinsic regrowth mechanisms of residual myomas not visible to the naked eye.

Our models were not sufficiently accurate to predict the time of myoma recurrence, mainly because the cases in this study were limited to a year's follow‐up, and each patient had follow‐up at only one specific timepoint. However, our results suggest that it is possible to predict whether residual myomas will have regrown by different timepoints. The inclusion of cases with longer follow‐up times in future studies will help to improve the correlation between residual myoma regrowth and the time variable.

Our study has several limitations. First, enrolled patients were followed for 1 year. Thus, records of longer follow‐up times are lacking. Some patients may have experienced residual myoma regrowth beyond the study period, limiting the model's applicability. Second, we selected T2WI for radiomics analysis to minimize the variability of acquisition parameters. However, this restriction may have resulted in less accurate prediction results; multimodal studies may improve accuracy further. Third, we chose only one feature selection method (LASSO) and one classifier (SVM). A combination of multiple classifiers and feature selection may yield a model with better prediction performance.

In conclusion, we developed and validated a combined prediction model based on T2WI radiomics through machine learning combined with clinicoradiological features. The model can predict effectively whether residual myoma recurrence occurs within 1 year after HIFU ablation of uterine myomas. It can serve as an accurate and convenient reference for clinical decision‐making, providing a basis for the early customization of chronic disease management plans after HIFU ablation in patients with uterine myomas.

## Supporting information


**Appendix S1** Formula for least absolute shrinkage and selection operator (LASSO)‐derived Rad‐scores

**Figure S1** Diagram summarizing study workflow. (a) Patient enrolment process and study cohorts. (b) Data collection. (c) Radiomics analysis, machine learning modeling and evaluation.

**Figure S2** Receiver‐operating‐characteristics curves and 95% CIs for prediction of residual uterine myoma regrowth after high‐intensity focused ultrasound ablation in training cohort, internal test cohort and external test cohort, using radiomics, clinic–radiological and combined radiomics–clinical models.

**Table S1** Feature extraction configuration following guidelines of the Image Biomarker Standardization Initiative (IBSI)
**Table S2** Extracted image features, classified into eight categories
**Table S3** Comparison of Rad‐scores between patients with and those without regrowth of uterine myoma after high‐intensity focused ultrasound ablation

## Data Availability

The data that support the findings of this study are available from the corresponding author upon reasonable request.

## References

[1] Liu Y , Wu X , Wu A , Gong C , Wang Z , Zhang L . Ultrasound‐guided high intensity focused ultrasound ablation for uterine fibroids: long‐term outcomes and factors affecting local recurrence. Int J Hyperthermia 2021; 38: 1341–1348. 34486914 10.1080/02656736.2021.1973585

[2] Ludovisi M , Moro F , Pasciuto T , Di Noi S , Giunchi S , Savelli L , Pascual MA , Sladkevicius P , Alcazar JL , Franchi D , Mancari R , Moruzzi MC , Jurkovic D , Chiappa V , Guerriero S , Exacoustos C , Epstein E , Fruhauf F , Fischerova D , Fruscio R , Ciccarone F , Zannoni GF , Scambia G , Valentin L , Testa AC . Imaging in gynecological disease (15): clinical and ultrasound characteristics of uterine sarcoma. Ultrasound Obstet Gynecol 2019; 54: 676–687. 30908820 10.1002/uog.20270

[3] Nieuwenhuis L , Keizer A , Stoelinga B , Twisk J , Hehenkamp W , Brölmann H , Huirne J . Fibroid vascularisation assessed with three‐dimensional power Doppler ultrasound is a predictor for uterine fibroid growth: a prospective cohort study. BJOG 2018; 125: 577–584. 28211610 10.1111/1471-0528.14608

[4] Hijnen N , Elevelt A , Grüll H . Stability and trapping of magnetic resonance imaging contrast agents during high‐intensity focused ultrasound ablation therapy. Invest Radiol 2013; 48: 517–524. 23695082 10.1097/RLI.0b013e31829aae98

[5] Kumar V , Gu Y , Basu S , Berglund A , Eschrich S , Schabath M , Forster K , Aerts H , Dekker A , Fenstermacher D , Goldgof D , Hall L , Lambin P , Balagurunathan Y , Gatenby R , Gillies R . Radiomics: the process and the challenges. Magn Reson Imaging 2012; 30: 1234–1248. 22898692 10.1016/j.mri.2012.06.010 PMC3563280

[6] Lambin P , Rios‐Velazquez E , Leijenaar R , Carvalho S , van Stiphout R , Granton P , Zegers C , Gillies R , Boellard R , Dekker A , Aerts H . Radiomics: extracting more information from medical images using advanced feature analysis. Eur J Cancer 2012; 48: 441–446. 22257792 10.1016/j.ejca.2011.11.036 PMC4533986

[7] Wang T , Gong J , Li Q , Chu C , Shen W , Peng W , Gu Y , Li W . A combined radiomics and clinical variables model for prediction of malignancy in T2 hyperintense uterine mesenchymal tumors on MRI. Eur Radiol 2021; 31: 6125–6135. 33486606 10.1007/s00330-020-07678-9

[8] Zheng Y , Chen L , Liu M , Wu J , Yu R , Lv F . Nonenhanced MRI‐based radiomics model for preoperative prediction of nonperfused volume ratio for high‐intensity focused ultrasound ablation of uterine leiomyomas. Int J Hyperthermia 2021; 38: 1349–1358. 34486913 10.1080/02656736.2021.1972170

[9] Tustison N , Avants B , Cook P , Zheng Y , Egan A , Yushkevich P , Gee J . N4ITK: improved N3 bias correction. IEEE Trans Med Imaging 2010; 29: 1310–1320. 20378467 10.1109/TMI.2010.2046908 PMC3071855

[10] Zwanenburg A , Vallières M , Abdalah M , Aerts H , Andrearczyk V , Apte A , Ashrafinia S , Bakas S , Beukinga R , Boellaard R , Bogowicz M , Boldrini L , Buvat I , Cook G , Davatzikos C , Depeursinge A , Desseroit M , Dinapoli N , Dinh C , Echegaray S , El Naqa I , Fedorov A , Gatta R , Gillies R , Goh V , Götz M , Guckenberger M , Ha S , Hatt M , Isensee F , Lambin P , Leger S , Leijenaar R , Lenkowicz J , Lippert F , Losnegård A , Maier‐Hein K , Morin O , Müller H , Napel S , Nioche C , Orlhac F , Pati S , Pfaehler E , Rahmim A , Rao A , Scherer J , Siddique M , Sijtsema N , Socarras Fernandez J , Spezi E , Steenbakkers R , Tanadini‐Lang S , Thorwarth D , Troost E , Upadhaya T , Valentini V , van Dijk L , van Griethuysen J , van Velden F , Whybra P , Richter C , Löck S . The Image Biomarker Standardization Initiative: Standardized Quantitative Radiomics for High‐Throughput Image‐based Phenotyping. Radiology 2020; 295: 328–338. 32154773 10.1148/radiol.2020191145 PMC7193906

[11] Huang X , Yu D , Zou M , Wang L , Xing H , Wang Z . The effect of exercise on high‐intensity focused ultrasound treatment efficacy in uterine fibroids and adenomyosis: a retrospective study. BJOG 2017; 124 (Suppl 3): 46–52. 28856860 10.1111/1471-0528.14748

[12] Gorny K , Borah B , Brown D , Woodrum D , Stewart E , Hesley G . Incidence of additional treatments in women treated with MR‐guided focused US for symptomatic uterine fibroids: review of 138 patients with an average follow‐up of 2.8 years. J Vasc Interv Radiol 2014; 25: 1506–1512. 24998103 10.1016/j.jvir.2014.05.012

[13] Kennedy J. High‐intensity focused ultrasound in the treatment of solid tumours. Nature Rev Cancer 2005; 5: 321–327. 15776004 10.1038/nrc1591

[14] Kim YS . Clinical application of high‐intensity focused ultrasound ablation for uterine fibroids. Biomed Eng Lett 2017; 7: 99–105. 30603156 10.1007/s13534-017-0012-9 PMC6208477

[15] Huang H , Ran J , Xiao Z , Ou L , Li X , Xu J , Wang Q , Wang Z , Li F . Reasons for different therapeutic effects of high‐intensity focused ultrasound ablation on excised uterine fibroids with different signal intensities on T2‐weighted MRI: a study of histopathological characteristics. Int J Hyperthermia 2019; 36: 477–484. 30915864 10.1080/02656736.2019.1592242

[16] Stewart E. Uterine fibroids. Lancet 2001; 357: 293–298. 11214143 10.1016/S0140-6736(00)03622-9

[17] Lambin P , Leijenaar R , Deist T , Peerlings J , de Jong E , van Timmeren J , Sanduleanu S , Larue R , Even A , Jochems A , van Wijk Y , Woodruff H , van Soest J , Lustberg T , Roelofs E , van Elmpt W , Dekker A , Mottaghy F , Wildberger J , Walsh S . Radiomics: the bridge between medical imaging and personalized medicine. Nat Rev Clin Oncol 2017; 14: 749–762. 28975929 10.1038/nrclinonc.2017.141

[18] Xie H , Hu J , Zhang X , Ma S , Liu Y , Wang X . Preliminary utilization of radiomics in differentiating uterine sarcoma from atypical leiomyoma: Comparison on diagnostic efficacy of MRI features and radiomic features. Eur J Radiol 2019; 115: 39–45. 31084757 10.1016/j.ejrad.2019.04.004

[19] Wei C , Li N , Shi B , Wang C , Wu Y , Lin T , Chen Y , Ge Y , Yu Y , Dong J . The predictive value of conventional MRI combined with radiomics in the immediate ablation rate of HIFU treatment for uterine fibroids. Int J Hyperthermia 2022; 39: 475–484. 35271784 10.1080/02656736.2022.2046182

[20] Nielsen B , Albregtsen F , Danielsen H . Statistical nuclear texture analysis in cancer research: a review of methods and applications. Crit Rev Oncog 2008; 14: 89–164. 19409060 10.1615/critrevoncog.v14.i2-3.10

[21] Dasarathy BV , Holder EB . Image characterizations based on joint gray level—run length distributions. Pattern Recognit Lett 1991; 12: 497–502.

[22] Lucia F , Visvikis D , Desseroit M , Miranda O , Malhaire J , Robin P , Pradier O , Hatt M , Schick U . Prediction of outcome using pretreatment F‐FDG PET/CT and MRI radiomics in locally advanced cervical cancer treated with chemoradiotherapy. Eur J Nucl Med Mol Imaging 2018; 45: 768–786. 29222685 10.1007/s00259-017-3898-7

[23] Park H , Park B , Park S , Choi S , Rhee H , Park J , Cho E , Yeom S , Park S , Park M , Lee S . Preoperative prediction of postsurgical outcomes in mass‐forming intrahepatic cholangiocarcinoma based on clinical, radiologic, and radiomics features. Eur Radiol 2021; 31: 8638–8648. 33890153 10.1007/s00330-021-07926-6

[24] Alfieri S , Romanò R , Bologna M , Calareso G , Corino V , Mirabile A , Ferri A , Bellanti L , Poli T , Marcantoni A , Grosso E , Tarsitano A , Battaglia S , Blengio F , De Martino I , Valerini S , Vecchio S , Richetti A , Deantonio L , Martucci F , Grammatica A , Ravanelli M , Ibrahim T , Caruso D , Locati L , Orlandi E , Bossi P , Mainardi L , Licitra L . Prognostic role of pre‐treatment magnetic resonance imaging (MRI)‐based radiomic analysis in effectively cured head and neck squamous cell carcinoma (HNSCC) patients. Acta Oncol 2021; 60: 1192–1200. 34038324 10.1080/0284186X.2021.1924401

[25] Sieren J , Smith A , Thiesse J , Namati E , Hoffman E , Kline J , McLennan G . Exploration of the volumetric composition of human lung cancer nodules in correlated histopathology and computed tomography. Lung Cancer 2011; 74: 61–68. 21371772 10.1016/j.lungcan.2011.01.023 PMC3129434

[26] Hocquelet A , Auriac T , Perier C , Dromain C , Meyer M , Pinaquy J , Denys A , Trillaud H , Denis De Senneville B , Vendrely V . Pre‐treatment magnetic resonance‐based texture features as potential imaging biomarkers for predicting event free survival in anal cancer treated by chemoradiotherapy. Eur Radiol 2018; 28: 2801–2811. 29404766 10.1007/s00330-017-5284-z

[27] Zhang J , Yang C , Gong C , Zhou Y , Li C , Li F . Magnetic resonance imaging parameter‐based machine learning for prognosis prediction of high‐intensity focused ultrasound ablation of uterine fibroids. Int J Hyperthermia 2022; 39: 835–846. 35764325 10.1080/02656736.2022.2090622

[28] Munro M , Critchley H , Broder M , Fraser I . FIGO classification system (PALM‐COEIN) for causes of abnormal uterine bleeding in nongravid women of reproductive age. Int J Gynaecol Obstet 2011; 113: 3–13. 21345435 10.1016/j.ijgo.2010.11.011

