# Supplementary material for: Prediction using T2‐weighted magnetic resonance imaging‐based radiomics of residual uterine myoma regrowth after high‐intensity focused ultrasound ablation
Source: Ultrasound Obstet Gynecol. 2022 Nov 1;60(5):681–92. doi: 10.1002/uog.26053 (PMC9828488; doi:10.1002/uog.26053)
Supplement: Supplementary file 1 — Appendix S1 Formula for least absolute shrinkage and selection operator (LASSO)‐derived Rad‐scores [file UOG-60-681-s001.docx]

**Appendix S1** Formula for least absolute shrinkage and selection operator (LASSO)-derived Rad-scores

$$Rad-score=original_{glrlm_{ShortRunHighGrayLevelEmphasis}}\times\left( -0.085493 \right)+original_{gldm_{DependenceVariance}}\times\left( -0.038819 \right)+wavelet_{LLH_{firstorder_{InterquartileRange}}}\times0.02828+wavelet_{LLH_{glrlm_{LongRunEmphasis}}}\times\left( -0.033433 \right)+wavelet_{LLH_{glrlm_{ShortRunLowGrayLevelEmphasis}}}\times\left( -0.014967 \right)+wavelet_{LHH_{glszm_{ZoneVariance}}}\times0.01648+wavelet_{HLL_{firstorder_{Maximum}}}\times0.03891+wavelet_{HLL_{firstorder_{Uniformity}}}\times0.05676+wavelet_{HLH_{glcm_{ClusterProminence}}}\times0.049487+wavelet_{HHL_{firstorder_{Minimum}}}\times\left( -0.025239 \right)+wavelet_{HHL_{glcm_{MCC}}}\times\left( -0.026839 \right)+wavelet_{HHL_{glcm_{Autocorrelation}}}\times0.039756+wavelet_{LLL_{firstorder_{InterquartileRange}}}\times\left( -0.013902 \right)+wavelet_{LLL_{firstorder_{Skewness}}}\times\left( -0.034776 \right)+wavelet_{LLL_{glrlm_{ShortRunLowGrayLevelEmphasis}}}\times0.025194+wavelet_{LLL_{glszm_{LargeAreaLowGrayLevelEmphasis}}}\times\left( -0.003910 \right)+wavelet_{LLL_{glszm_{SizeZoneNonUniformity}}}\times0.00170+0.45679012$$
