# Supplementary figures and images for: Prediction using T2‐weighted magnetic resonance imaging‐based radiomics of residual uterine myoma regrowth after high‐intensity focused ultrasound ablation
Source: Ultrasound Obstet Gynecol. 2022 Nov 1;60(5):681–92. doi: 10.1002/uog.26053 (PMC9828488; doi:10.1002/uog.26053)

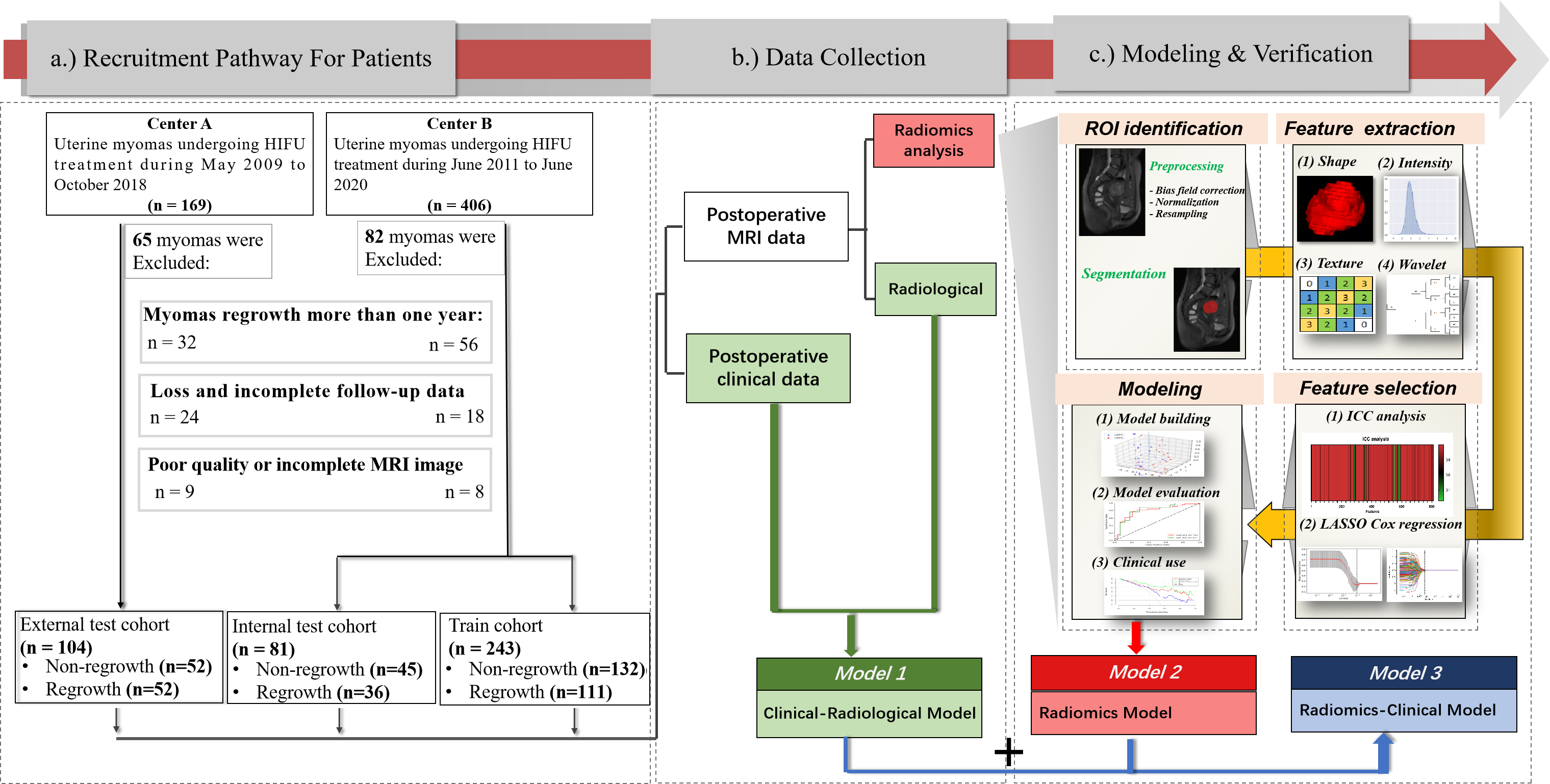

Supplement: Supplementary file 2 — Figure S1 Diagram summarizing study workflow. (a) Patient enrolment process and study cohorts. (b) Data collection. (c) Radiomics analysis, machine learning modeling and evaluation. [file UOG-60-681-s004.tif]
