# Supplementary material for: Prediction using T2‐weighted magnetic resonance imaging‐based radiomics of residual uterine myoma regrowth after high‐intensity focused ultrasound ablation
Source: Ultrasound Obstet Gynecol. 2022 Nov 1;60(5):681–92. doi: 10.1002/uog.26053 (PMC9828488; doi:10.1002/uog.26053)
Supplement: Supplementary file 3 — Figure S2 Receiver‐operating‐characteristics curves and 95% CIs for prediction of residual uterine myoma regrowth after high‐intensity focused ultrasound ablation in training cohort, internal test cohort and external test cohort, using radiomics, clinic–radiological and combined radiomics–clinical models. [file UOG-60-681-s003.docx]

| **Figure S2** Receiver-operating-characteristics curves and 95% CIs for prediction of residual uterine myoma regrowth after high-intensity focused ultrasound ablation in training cohort, internal test cohort and external test cohort, using radiomics, clinic–radiological and combined radiomics–clinical models. AUC, area under the curve. |
| --- |

| Model  Datasets | *Radiomics* | *Clinicoradiological* | *Combined radiomics-clinical* |
| --- | --- | --- | --- |
| Training cohort | 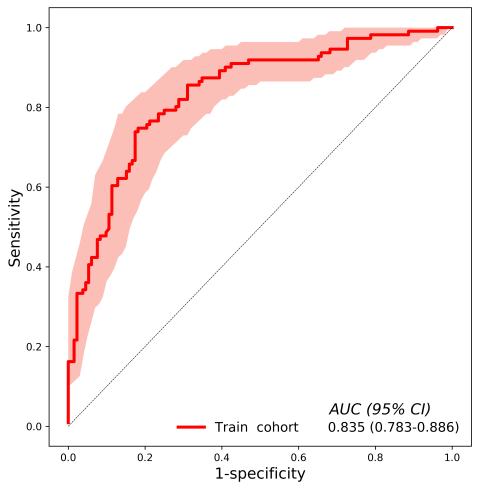 | 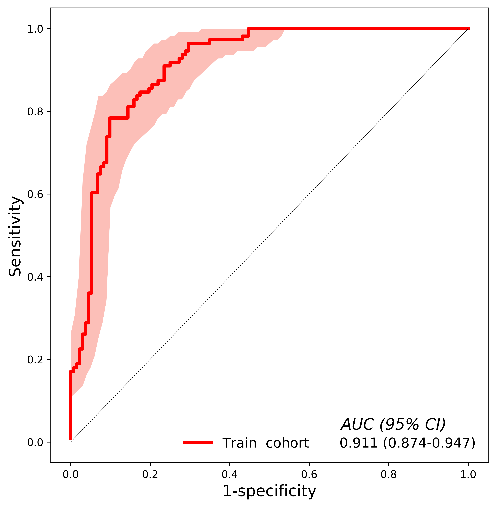 | 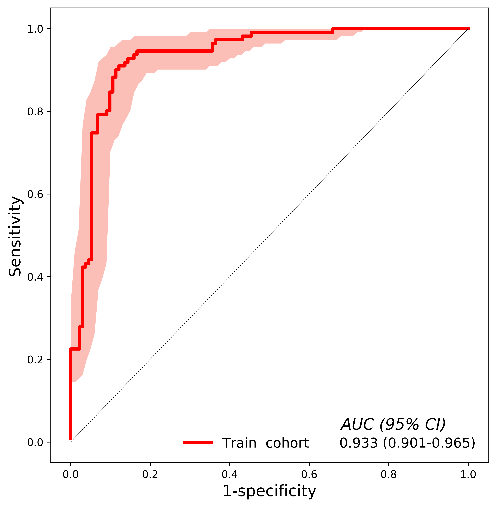 |
| Internal test cohort | 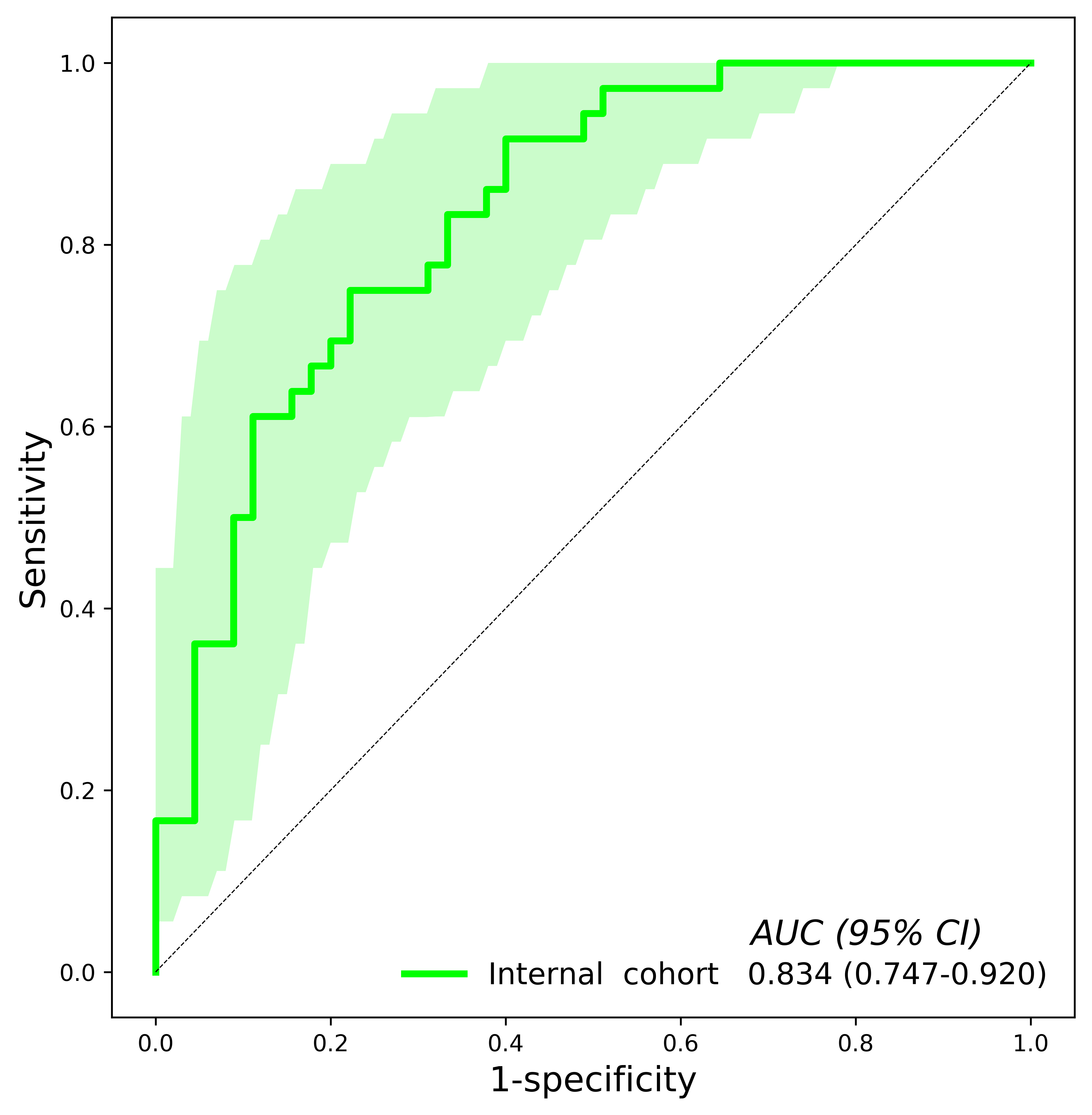 | 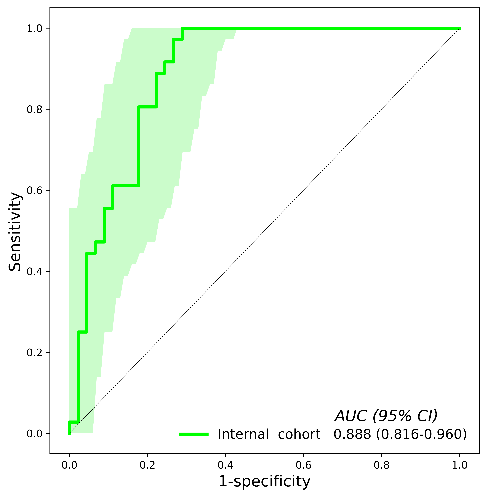 | 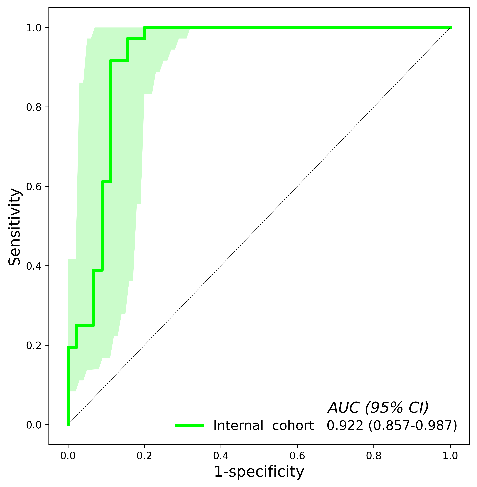 |
| External test cohort | 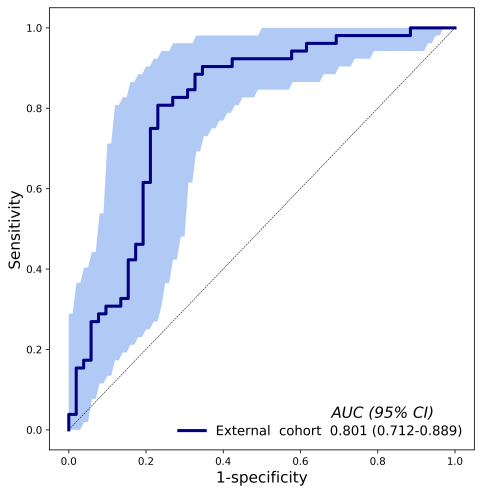 | 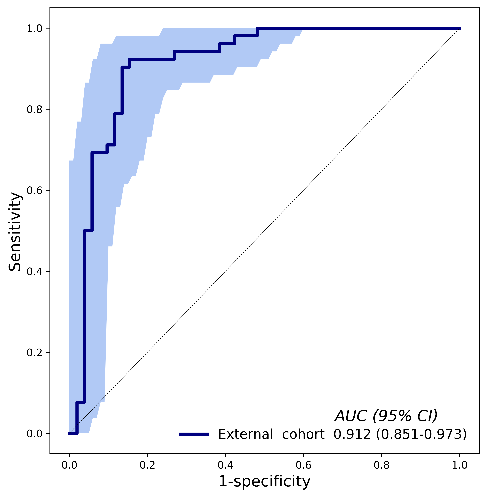 | 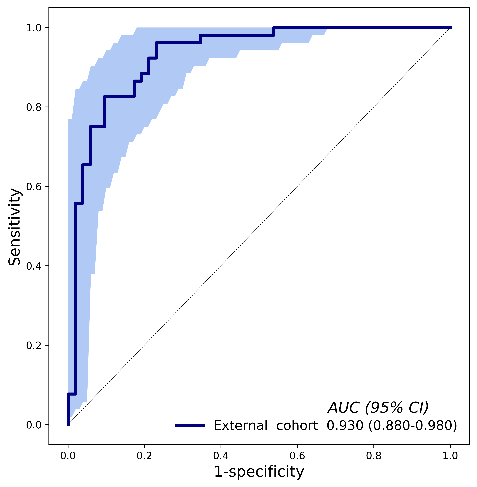 |
