# Supplementary material for: Prediction using T2‐weighted magnetic resonance imaging‐based radiomics of residual uterine myoma regrowth after high‐intensity focused ultrasound ablation
Source: Ultrasound Obstet Gynecol. 2022 Nov 1;60(5):681–92. doi: 10.1002/uog.26053 (PMC9828488; doi:10.1002/uog.26053)
Supplement: Supplementary file 4 — Table S1 Feature extraction configuration following guidelines of the Image Biomarker Standardization Initiative (IBSI) Table S2 Extracted image features, classified into eight categories Table S3 Comparison of Rad‐scores between patients with and those without regrowth of uterine myoma after high‐intensity focused ultrasound ablation [file UOG-60-681-s002.docx]

**Table S1** Feature extraction configuration following guidelines of the Image Biomarker Standardization Initiative (IBSI)

| Parameter |  | Config | | | |
| --- | --- | --- | --- | --- | --- |
| Slice-wise or single-volume (3D) |  | 3D | | | |
| Interpolation |  |  | | | |
|  | Resampled voxel spacing (mm) | 1 × 1 × 1 | | | |
|  | ROI interpolation method | B-spline | | | |
| Re-segmentation |  |  | | | |
|  | Range | [μ －3σ, μ ＋3σ] | | | |
|  | Outlier filtering | no | | | |
| Discretisation |  |  | | | |
|  | Texture and intensity-based statistics features | BinWidth: 25 | | | |
| Feature parameters |  | **Distance** | **Count** | **ROI mask** | |
|  |  |  |  | Morph. | Int. |
|  | Morphology | **×** | **14** | **√** | **×** |
|  | Intensity-based statistics | **×** | **18** | **×** | **√** |
|  | GLCM | **1** | **24** | **×** | **√** |
|  | GLRLM | **1** | **16** | **×** | **√** |
|  | GLSZM | **1** | **16** | **×** | **√** |
|  | GLDM | **1** | **14** | **×** | **√** |
|  | NGTDM | **1** | **5** | **×** | **√** |

μ, mean of ROI; σ, standard deviation of ROI; Int, intensity mask; Morph, morphological mask; ROI, region of interest

**Table S2** Extracted image features, classified into eight categories

| 1. First-order statistics (18 features) | Energy; Total Energy; Entropy; Minimum; 10th percentile; 90th percentile; Maximum; Mean; Median; Interquartile Range; Range; Mean Absolute Deviation (MAD); Robust Mean Absolute Deviation (rMAD); Root Mean Squared (RMS); Skewness; Kurtosis; Variance; Uniformity |
| --- | --- |
| 2. Shape-based (3D) (14 features) | Mesh Volume; Voxel Volume; Surface Area; Surface Area to Volume ratio; Sphericity; Maximum 3D diameter; Maximum 2D diameter (Slice); Maximum 2D diameter (Column); Maximum 2D diameter (Row); Major Axis Length; Minor Axis Length; Least Axis Length; Elongation; Flatness |
| 3. Gray-level co-occurrence matrix (GLCM) (24 features) | Autocorrelation; Joint Average; Cluster Prominence; Cluster Shade; Cluster Tendency; Contrast; Correlation; Difference Average; Difference Entropy; Difference Variance; Joint Energy; Joint Entropy; Informational Measure of Correlation (IMC) 1; Informational Measure of Correlation (IMC) 2; Inverse Difference Moment (IDM); Maximal Correlation Coefficient (MCC); Inverse Difference Moment Normalized (IDMN); Inverse Difference (ID); Inverse Difference Normalized (IDN); Inverse Variance; Maximum Probability; Sum Average; Sum Entropy; Sum of Squares |
| 4. Gray-level size zone matrix (GLSZM) (16 features) | Short Run Emphasis (SRE); Long Run Emphasis (LRE); Gray Level Non-Uniformity (GLN); Gray Level Non-Uniformity Normalized (GLNN); Run Length Non-Uniformity (RLN); Run Length Non-Uniformity Normalized (RLNN); Run Percentage (RP); Gray Level Variance (GLV); Run Variance (RV); Run Entropy (RE); Low Gray Level Run Emphasis (LGLRE); High Gray Level Run Emphasis (HGLRE); Short Run Low Gray Level Emphasis (SRLGLE); Short Run High Gray Level Emphasis (SRHGLE); Long Run Low Gray Level Emphasis (LRLGLE); Long Run High Gray Level Emphasis (LRHGLE) |
| 5. Gray-level run-length matrix (GLRLM) (16 features) | Small Area Emphasis (SAE); Large Area Emphasis (LAE); Gray Level Non-Uniformity (GLN); Gray Level Non-Uniformity Normalized (GLNN); Size-Zone Non-Uniformity (SZN); Size-Zone Non-Uniformity Normalized (SZNN); Zone Percentage (ZP); Gray Level Variance (GLV); Zone Variance (ZV); Zone Entropy (ZE); Low Gray Level Zone Emphasis (LGLZE); High Gray Level Zone Emphasis (HGLZE); Small Area Low Gray Level Emphasis (SALGLE); Small Area High Gray Level Emphasis (SAHGLE); Large Area Low Gray Level Emphasis (LALGLE); Large Area High Gray Level Emphasis (LAHGLE) |
| 6. Gray-level dependence matrix (GLDM) (14 features) | Small Dependence Emphasis (SDE); Large Dependence Emphasis (LDE); Gray Level Non-Uniformity (GLN); Dependence Non-Uniformity (DN); Dependence Non-Uniformity Normalized (DNN); Gray Level Variance (GLV); Dependence Variance (DV); Dependence Entropy (DE); Low Gray Level Emphasis (LGLE); High Gray Level Emphasis (HGLE); Small Dependence Low Gray Level Emphasis (SDLGLE); Small Dependence High Gray Level Emphasis (SDHGLE); Large Dependence Low Gray Level Emphasis (LDLGLE); Large Dependence High Gray Level Emphasis (LDHGLE) |
| 7. Neighbouring gray tone difference matrix (NGTDM) (5 features) | Coarseness; Contrast; Busyness; Complexity; Strength |
| 8. Wavelet features (744 features)* | wavelet-LLH; wavelet-LHL; wavelet-LHH; wavelet-HLH; wavelet-HLL; wavelet-LLL; wavelet-HHL; wavelet-HHH |

*Eight filters were applied to the original images, yielding derived images for each patient.All categories of features except shape features were recalculated on the derived images.

**Table S3** Comparison of Rad-scores between patients with and those without regrowth of uterine myoma after high-intensity focused ultrasound ablation

|  | Non-regrowth | Regrowth | p-value |
| --- | --- | --- | --- |
| Training set | 0.366±0.141 | 0.556±0.159 | **< 0.001*** |
| Internal test set | 0.364±0.139 | 0.521±0.133 | **< 0.001*** |
| External test set | 0.469±0.147 | 0.617±0.143 | **< 0.001*** |
| The independent samples *t* test was used to compare the differences in Rad-scores between the two groups, *p < 0.05 means statistical significance. | | | |
